# Effectiveness of the various revascularization techniques in multivessel coronary artery disease: a systematic review with network meta-analysis

**DOI:** 10.1080/07853890.2025.2566878

**Published:** 2025-09-30

**Authors:** I Komang Adhi Parama Harta, Putu Febry Krisna Pertiwi, I Gde Julia Arta, Ketut Putu Yasa

**Affiliations:** ^a^ Cardiothoracic and Vascular Surgery Division, Department of Surgery, Faculty of Medicine, Udayana University, Prof. Dr. I.G.N.G. Ngoerah General Hospital, Denpasar, Bali, Indonesia; ^b^ Faculty of Medicine, Udayana University, Denpasar, Indonesia; ^c^ Cardiothoracic and Vascular Surgery Residency Program, Faculty of Medicine, Udayana University, Denpasar, Indonesia

**Keywords:** Coronary revascularization, multivessels disease, off-pump coronary artery bypass (OPCAB), anaortic technique, network meta-analysis

## Abstract

**Background:**

Multivessel coronary artery disease (MVD) often requires revascularization. However, the effectiveness of various techniques in reducing stroke and achieving complete revascularization remains uncertain. This study aimed to address this gap by comparing key revascularization strategies in terms of early mortality, stroke, complete revascularization, postoperative atrial fibrillation (POAF), and renal failure.

**Methods:**

This study is a systematic review and network meta-analysis of 32 studies including 65,861 patients. Five revascularization techniques were compared: on-pump coronary artery bypass (ONCAB), off-pump coronary artery bypass (OPCAB), OPCAB with proximal anastomotic device (OPCAB-PAD), anaortic OPCAB (anOPCAB), and percutaneous coronary intervention (PCI). Odds ratios (ORs) with 95% confidence intervals (CIs) were calculated using a random effects model. Risk of bias was assessed using the RoB2 and ROBINS-I tools.

**Results:**

Compared to ONCAB, early mortality was significantly lower with anOPCAB (OR: 0.57, 95% CI: 0.44–0.73), OPCAB-PAD (OR: 0.61, 95% CI: 0.40–0.92), and OPCAB (OR: 0.64, 95% CI: 0.47–0.87). Stroke risk was lowest with anOPCAB (OR: 0.29, 95% CI: 0.21–0.40) and OPCAB-PAD (OR: 0.32, 95% CI: 0.21–0.49). All surgical techniques achieved significantly more complete revascularization than PCI. Both POAF and renal failure were significantly lower with anOPCAB compared to ONCAB (POAF: OR: 0.72, 95% CI: 0.59–0.89; renal failure: OR: 0.63, 95% CI: 0.46–0.86). No significant publication bias was detected for mortality and stroke, though funnel plot asymmetry was noted for revascularization.

**Conclusion:**

Off-pump techniques, particularly anOPCAB, significantly reduce stroke risk while achieving comparable revascularization success to ONCAB. PCI remains limited by incomplete revascularization, supporting its use primarily in patients at high surgical risk.

## Graphical Abstract

**Figure:**
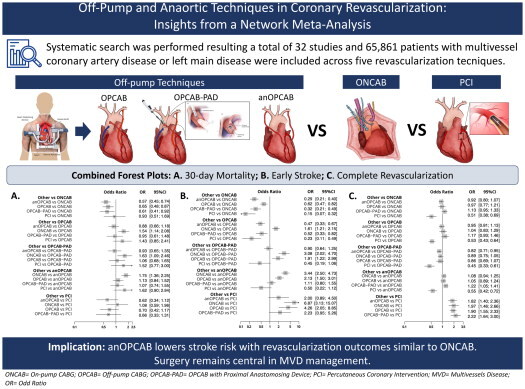
Network meta-analysis of 32 studies (65,861 patients) comparing off-pump (OPCAB, OPCAB-PAD, anOPCAB), ONCAB, and PCI in multivessel disease. Forest plots show early mortality, stroke, and complete revascularization.

## Introduction

Coronary artery disease (CAD) and its ischemic complications remain the leading global causes of death and disability-adjusted life years (DALYs), accounting for over 9 million deaths annually according to the Global Burden of Disease study [1,2]. Despite advances in preventive cardiology and widespread use of pharmacologic agents such as statins, antiplatelets, and antihypertensives, many patients especially those with multivessel or left main disease continue to experience ischemic events requiring more definitive interventions [3,4].

Contemporary guidelines from both the ACC/AHA and ESC support coronary revascularization in appropriately selected patients with chronic or acute coronary syndromes, particularly when high-risk anatomy or ongoing ischemia is present despite optimal medical therapy [5,6]. While pharmacologic management remains the foundation for prevention, revascularization whether surgical or percutaneous plays a critical role in improving long-term survival, reducing myocardial infarction, and enhancing quality of life in symptomatic patients with obstructive CAD.

Surgical coronary artery bypass grafting (CABG) using cardiopulmonary bypass, known as on-pump CABG (ONCAB), has traditionally been the gold standard due to its ability to achieve complete revascularization [7]. However, this technique involves aortic manipulation and cardiopulmonary bypass, both of which are associated with increased risks of stroke, renal injury, and cognitive dysfunction [8]. To mitigate these risks, less invasive surgical approaches have been developed, including off-pump CABG (OPCAB), OPCAB with proximal anastomotic devices (OPCAB-PAD), and anaortic OPCAB (anOPCAB), which aim to reduce or eliminate aortic manipulation altogether [9,10]. In parallel, percutaneous coronary intervention (PCI) has evolved as a less invasive alternative, though concerns remain about its limited ability to achieve complete revascularization in patients with complex or diffuse disease [11].

Although several pairwise meta-analyses have compared subsets of these techniques, the evidence remains fragmented. Conventional meta-analyses are limited when direct comparisons are lacking. Network meta-analysis (NMA), which combines both direct and indirect evidence across multiple interventions, offers a more robust framework for comparative effectiveness in the absence of head-to-head trials. However, to date, there has been no comprehensive NMA comparing all five revascularization strategies: ONCAB, OPCAB, OPCAB-PAD, anOPCAB, and PCI in patients with multivessel disease.

This study aims to address this gap by performing a network meta-analysis comparing these five revascularization techniques across five critical outcomes: early mortality, early stroke, complete revascularization, postoperative atrial fibrillation (POAF), and renal failure. While bleeding complications are an important consideration in revascularization strategies, this analysis focuses on ischemic and procedural outcomes to provide a focused assessment of each technique’s efficacy and neurologic safety.

## Methods

### Study design

This study is a systematic review and network meta-analysis conducted in accordance with the PRISMA (Preferred Reporting Items for Systematic Reviews and Meta-Analyses) 2020 guidelines [12]. This study aimed to compare the effectiveness and safety of five coronary revascularization techniques, anaortic OPCAB (anOPCAB), OPCAB with proximal anastomotic device (OPCAB-PAD), standard OPCAB, ONCAB, and PCI in patients with multivessel coronary artery disease, focusing on five key outcomes: early mortality, early stroke, complete revascularization, postoperative atrial fibrillation (POAF), and renal failure. The study protocol was registered with PROSPERO (CRD42024586444).

### Study selection and data extraction

A comprehensive systematic literature search was performed in four major databases: PubMed, Embase, and ScienceDirect. The search was conducted from database inception to August 7, 2024. No lower date limit was applied to ensure comprehensive coverage of all relevant studies. The full search strings for each database are provided in Appendix 1. Manual backward citation tracking of key studies and relevant reviews was also performed. No language restrictions were applied. We included only full-text, peer-reviewed articles and explicitly excluded unpublished abstracts, conference proceedings, and non-peer-reviewed reports to ensure the reliability and completeness of extracted data. Title and abstract screening were conducted independently by two reviewers (IKAPH, PFKP), followed by full-text screening using predefined inclusion and exclusion criteria. Disagreements were resolved through discussion with a third author (IGJA). A fourth author (KPY) reviewed the final selections to ensure consensus and completeness.

### Inclusion and exclusion criteria

Inclusion criteria for this study encompassed randomized controlled trials (RCTs) or observational studies that compared at least two of the following techniques: anaortic off-pump coronary artery bypass (anOPCAB), OPCAB with proximal anastomosis device (OPCAB-PAD), standard OPCAB, on-pump coronary artery bypass (ONCAB), or percutaneous coronary intervention (PCI). Additionally, studies were required to report data on at least one of the following outcomes: early mortality, early stroke, complete revascularization, postoperative atrial fibrillation (POAF), or renal failure, and involve adult patients (aged 18 years or older) with multivessel coronary artery disease or left main disease. Studies were excluded if they did not have an appropriate comparison, failed to report relevant outcomes, or were duplicate reports or studies with incomplete data.

### Definition of procedures

This study included five coronary revascularization techniques: ONCAB, OPCAB, OPCAB-PAD, anOPCAB, and PCI. ONCAB (on-pump coronary artery bypass) involves cardiopulmonary bypass with aortic cross-clamping to perform coronary anastomoses. OPCAB (off-pump coronary artery bypass) is conducted on a beating heart without cardiopulmonary bypass, typically involving partial clamping of the ascending aorta for proximal anastomoses. OPCAB-PAD utilizes a mechanical device, such as the Heartstring, Enclose II, or PAS-Port, to perform proximal anastomoses without aortic side clamping. anOPCAB eliminates all aortic manipulation by using *in situ* grafting strategies (e.g. bilateral internal mammary arteries and T- or Y-grafts) without clamping. PCI (percutaneous coronary intervention) refers to catheter-based techniques to revascularize stenosed coronary arteries, typically *via* balloon angioplasty and stent deployment. In the included studies, PCI procedures involved both bare-metal and drug-eluting stents, depending on the time of study and operator preference. Most studies applied guideline-directed dual antiplatelet therapy (DAPT) post-procedure (e.g. aspirin plus clopidogrel or ticagrelor), although specific drug regimens were variably reported and not analyzed in this meta-analysis. Information on adjunctive pharmacologic management (e.g. anticoagulation or lipid-lowering therapy) was reported inconsistently and was therefore not incorporated in comparative analysis.

### Outcomes

The primary outcomes of interest were early mortality, early stroke, and complete revascularization. Early mortality defined as mortality within 30 days of the procedure or during the index hospitalization. Early stroke, defined as the occurrence of any new focal neurological deficit or brain injury within 30 days after the procedure, confirmed by clinical examination and imaging (CT or MRI), as well as transient ischemic attacks (TIA) that result in lasting neurological impairment (stroke occurring within 30 days of the procedure). Complete revascularization defined as the successful restoration of blood flow to all intended target vessels, based on angiographic findings or intraoperative assessment. POAF defined as AF in the postoperative setting requiring treatment with rate or rhythm control agents, with or without anticoagulation. Postoperative renal failure was defined as the new onset of dialysis requirement or a postoperative serum creatinine level that was at least twice the preoperative baseline and equal to or greater than 2.0 mg/dL. Although we acknowledge the clinical importance of additional events such as stent thrombosis, myocardial infarction, composite cardiovascular outcomes, and bleeding complications, these endpoints were not consistently reported across the included studies. Consequently, they were not included in this analysis to maintain methodological consistency and reduce the risk of bias from incomplete or heterogeneous outcome definitions.

### Quality assessment

To evaluate the methodological quality of included studies, we used the RoB 2 tool for randomized controlled trials (RCTs) and the ROBINS-I tool for observational studies, as recommended by the Cochrane Collaboration (Sterne et al. 2016, 2019). The RoB 2 tool assessed five domains of bias: the randomization process, deviations from intended interventions, missing outcome data, measurement of the outcome, and selection of the reported result. Each domain was rated as having low risk, some concerns, or high risk of bias. For observational studies, the ROBINS-I tool evaluated seven domains: confounding, selection of participants, classification of interventions, deviations from intended interventions, missing data, measurement of outcomes, and selection of the reported result. These were rated as low, moderate, serious, or critical risk of bias. The overall risk of bias judgment for each study was determined by the highest level of risk identified across domains.

### Statistical analysis

A frequentist network meta-analysis was performed using a random effects model in the ‘netmeta’ package in R version 4.3.2 (R Core Team, Vienna, Austria). The analysis synthesized both direct and indirect comparisons across the five revascularization techniques: anaortic off-pump coronary artery bypass (anOPCAB), OPCAB with proximal anastomotic device (OPCAB-PAD), standard off-pump coronary artery bypass (OPCAB), on-pump coronary artery bypass (ONCAB), and percutaneous coronary intervention (PCI). Odds ratios (ORs) with 95% confidence intervals (CI) were calculated for each pairwise comparison, using a random effects model to account for variability between studies. This approach allowed for the pooling of results across multiple interventions, even when direct comparisons were lacking. In addition to estimating pooled odds ratios, we ranked the interventions using P-scores, a measure derived from the point estimates and standard errors of treatment effects under a frequentist framework. The P-score ranges from 0 (worst) to 1 (best), with higher values indicating higher efficacy of an intervention compared to others. P-scores were computed for each primary outcome using the netmeta package in R. Heterogeneity was quantified using tau^2^ (between-study variance) and I^2^ statistics. An *I*^2^ value of greater than 50% was considered indicative of significant heterogeneity. Funnel plots and Egger’s regression test were used to assess the presence of publication bias.

## Results

**Figure 1.**
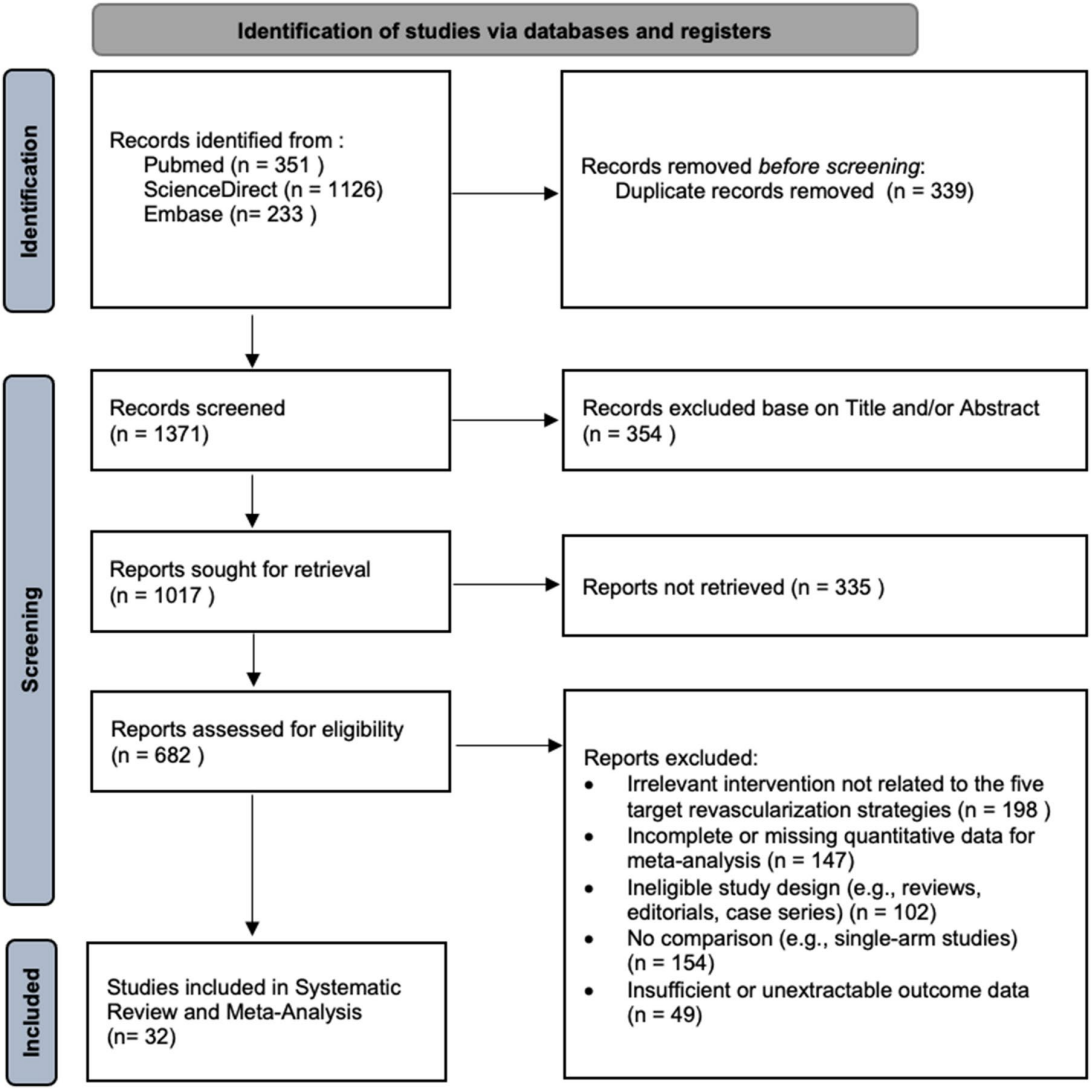
PRISMA flow diagram.

The systematic search yielded a total of 1477 records. After selection process, a total of 32 studies were met the inclusion criteria and included in this review. Of the 32 included studies, 5 were randomized controlled trials (RCTs) and 27 were observational studies. The selection process is illustrated in the PRISMA flow diagram (Figure 1). A total of 65,861 patients were included in this study, distributed among the following groups: anOPCAB (19,168), OPCAB-PAD (6794), OPCAB (10,159), ONCAB (20,141), and PCI (8402). The baseline characteristics of these patients are summarized in Table 1.

**Table 1.** Baseline characteristics of included studies.

Sudy	Year	Design	Comparison	Population (N)	Mean age, (SD)	HTN (%)	DM (%)	Dysli-pide-mia	Renal insufficiency (%)	CVA (%)	3VD (%)	LMD (%)
Calafiore	2002	Retrospective	anOPCAB	1533	NR	NR	NR	NR	NR	NR	NR	NR
			OPCAB	460	NR	NR	NR	NR	NR	NR	NR	NR
			ONCAB	2233	NR	NR	NR	NR	NR	NR	NR	NR
Albert	2018	Retrospective	anOPCAB	4485	NR	NR	NR	NR	NR	NR	NR	NR
			ONCAB	8794	NR	NR	NR	NR	NR	NR	NR	NR
Emmert	2012	Retrospective	anOPCAB	272	64 (11)	48.2	25.7	76.9	0.7	1.8	100	32.4
			ONCAB	155	62 (9)	61.3	20.6	75.5	1.9	0	100	29.7
Furmica	2017	Retrospective	anOPCAB	363	69.67 (11.15)	75.2	26.4	39.7	11	5.8	28.1	17.6
			OPCAB-PAD	282	71.13 (8.69)	70.6	37.2	35.8	16.3	9.9	36.3	32.3
Furukawa	2017	Prospective	anOPCAB	935	64.5 (9.5)	82.6	19	83.1	0.5	3.1	79.5	NR
			OPCAB-PAD	935	64.8 (9.9)	82	17.9	82.7	0.2	2.6	77.4	NR
			ONCAB	935	64.9 (9.4)	83.9	18.8	82.7	0.4	3.3	77.5	NR
Ushioda	2024	Retrospective	anOPCAB	640	64.5 (8.5)	98.6	49.2	98.6	13.9	7.19	78.6	41.7
			OPCAB	640	64.7 (8.3)	98.4	47.7	98.1	11.6	7.03	79.2	41.7
Vallely	2008	Prospective	anOPCAB	1201	67.6 (NR)	69.1	25	NR	4.7	NR	37.3	60.4
			OPCAB	557	67.6 (NR)	67.1	23.2	NR	4.3	NR	35.2	56
Misfeld	2010	Retrospective	anOPCAB	1346	67.2 (10.7)	69.8	25.4	72.6	4.2	4.7	NR	NR
			OPCAB	600	67.2 (10.7)	68.2	25	72.2	5	4	NR	NR
			ONCAB	1753	66.3 (10.4)	65.1	27.2	72.2	3.5	2.9	NR	NR
Saito	2020	Retrospective	anOPCAB	5012	69.3 (10.2)	78.3	51.3	63.5	10.3	12.7	32.8	25.2
			OPCAB-PAD	5012	69.3 (9.8)	78.7	51.5	64.5	10.1	11.9	32.5	25.7
Kim	2002	Retrospective	anOPCAB	222	61 (9)	59	37.8	20.3	4.5	11.7	67.1	24.3
			OPCAB	123	63 (9)	61.8	43.9	24.4	6.5	13	77.2	27.6
			ONCAB	76	62 (8)	53.9	38.2	19.7	10.5	6.6	71.1	26.3
Patel	2002	Retrospective	anOPCAB	597	61 (NR)	50.9	18.1	83.1	3.5	6.9	60.3	16.3
			OPCAB	520	63 (NR)	46.4	13.9	78.8	2.1	7.1	64.2	18.5
			ONCAB	1210	62 (NR)	40.3	16.7	73.3	1.7	6.9	79.1	21.2
Leacche	2003	Retrospective	anOPCAB	84	62 (13)	46	18	79	2.5	17	31	30
			OPCAB	556	64 (10)	47	25	69	1.6	8.4	71.6	30
Kapetanakis	2004	Retrospective	anOPCAB	476	61.2 (11.3)	62.6	25	NR	1.7	0	NR	9.5
			OPCAB	2527	66.2 (10.7)	69.1	33.6	NR	2.1	0.3	NR	13.9
			ONCAB	4296	64.0 (10.4)	69.7	37.9	NR	1.9	0.1	NR	19.1
Lev-Ran	2005	Retrospective	anOPCAB	429	67.4 (11.5)	66	39.4	NR	5.8	9.6	93	28.2
			OPCAB	271	68.4 (10.9)	61.7	40	NR	13	8.5	93.7	29.3
Manabe	2009	Retrospective	anOPCAB	185	68.4 (8.8)	71.9	38.4	54.6	NR	10.8	40	NR
			OPCAB-PAD	109	70.9 (8.4)	75.2	38.5	57.8	NR	20.2	79.8	NR
			OPCAB	241	68.1 (9.1)	75.5	42.7	63.9	NR	9.1	80.9	NR
Matsuura	2013	Retrospective	anOPCAB	264	67.3 (8.0)	74.6	43.6	59.8	8.3	9.1	40.9	30.7
			OPCAB	72	68.9 (9.1)	65.3	43.1	61.1	5.6	5.6	61.1	47.2
Zayat	2011	Prospective randomized trial	OPCAB-PAD	29	62.3 (11.7)	96.5	46.4	93.1	10.3	10.34	NR	NR
			OPCAB	28	62.95 (11.15)	100	53.5	7.1	0	14.3	NR	NR
Kempfert	2008	Prospective randomized trial	OPCAB-PAD	51	74.5 (0.6)	NR	39.2	84.3	NR	NR	NR	NR
			OPCAB	48	75.51 (0.5)	NR	35.4	72.9	NR	NR	NR	NR
Bassano	2015	Retrospective	anOPCAB	143	67.4 (9.4)	88.1	41.3	NR	2.8	11.2	NR	30.1
			ONCAB	143	67.4 (9.0)	89.5	44.8	NR	2.8	10.5	NR	31.5
Ohira	2016	Retrospective	anOPCAB	523	67.4 (9.5)	60.4	45.7	47.6	12.8	27.9	NR	41.3
			OPCAB-PAD	376	68.4 (8.8)	74.2	55.9	56.4	16.5	34.6	NR	40.7
			ONCAB	152	64.3 (9.0)	49.7	40.4	40.4	6	13.5	NR	34.4
Borgermann	2012	Retospective	anOPCAB	394	69.3 (9.1)	82.2	22.8	NR	0.8	1	NR	25.1
			ONCAB	394	69.0 (8.9)	82.2	19.8	NR	0.3	1.8	NR	24.9
Szwed	2021	Randomized clinical trial	anOPCAB	64	65.2 (7.7)	82.8	10.9	54.7	3.1	NR	NR	NR
			OPCAB	64	66.3 (9.0)	81.3	9.4	67.2	4.7	NR	NR	NR
Briguori	2007	Retrospective	PCI	69	63 (9)	74	100	55	NR	NR	NR	NR
			OPCAB	149	66 (9)	80	100	66	NR	NR	NR	NR
Chieffo	2006	Retrospective	PCI	107	63.6 (10.3)	58.8	18.7	70	1.9	NR	NR	NR
			OPCAB	56	67.5 (9.7)	76	23.2	69	8.4	NR	NR	NR
Drenth	2002	Prospective randomized trial	PCI	51	61 (1.3)	33	18	45	NR	NR	NR	NR
			OPCAB	51	60 (1.6)	16	8	41	NR	NR	NR	NR
Eefting	2003	Prospective randomized trial	PCI	138	60.3 (9.1)	33	9	59	NR	1	NR	NR
			OPCAB	142	58.9 (10.0)	31	14	60	NR	2	NR	NR
Marui	2012	Retrospective	PCI	3877	68.3 (10.0)	72.5	42.6	50.4	4.3	15.7	37.7	4.3
			OPCAB	1381	68.6 (9.3)	75.2	46.8	57.1	5	26.9	66.2	31
Yamagata	2010	Prospective	PCI	92	70 (9)	91	100	82	NR	29	NR	NR
			OPCAB	116	67 (7)	92	100	84	NR	31	NR	NR
Yin	2015	Retrospective	PCI	106	61.67 (9.23)	66.9	21.7	44.34	NR	16	NR	100
			OPCAB	121	60.96 (6.8)	65.2	21.5	54.55	NR	11.6	NR	100
Jeong	2013	Retrospective	PCI	159	60.9 (9.9)	49.1	31.4	1.7	0	3.1	32.8	NR
			OPCAB	159	60.8 (10)	49.1	30.8	1.7	0	3.1	45.3	NR
Lee	2018	Retrospective	PCI	714	64.2 (10.5)	62.2	49.4	28.4	8.3	6.9	97.2	NR
			OPCAB	1227	64.0 (10.1)	60.2	47.8	28	7.5	7.3	97.1	NR
Mack	2008	Prospective	PCI	3089	63.5 (12.4)	74.3	32.8	67.3	4.4	4.5	NR	NR
			OPCAB	1247	63.6 (10.6)	78.8	35	54.3	4.9	7.1	NR	NR

Abbreviations: ONCAB: on-pump CABG, OPCAB: off-pump CABG, anOPCAB: anaortic OPCAB, OPCAB-PAD: OPCAB with proximal anastomotic device, NR: not reported, HTN: hypertension, DM: diabetes mellitus type 2, CVA: cerebrovascular accident, 3VD: 3 vessels disease, LMD: left main disease.

### Quality assessment

The quality assessment of included studies was conducted using two standardized tools. For the 27 observational studies, the ROBINS-I tool was used. Out of these, 18 studies (66.7%) were judged to have overall low risk of bias, while 9 studies (33.3%) were rated as having moderate risk of bias. None of the observational studies were rated as having serious or critical risk. Domain-level analysis indicated that most concerns were related to bias due to confounding, selection of participants, and missing data (Appendix 2.).

For the 5 randomized controlled trials (RCTs), the RoB 2 tool was used. Among them, 3 trials (60%) were judged to have low overall risk of bias, while 2 trials (40%) were rated as having some concerns, primarily in the domains of randomization process and selection of the reported result. No RCTs were rated as having high risk of bias. These findings indicate that the majority of studies included in the network meta-analysis are of acceptable quality, although attention should be paid to certain methodological limitations present in both observational and randomized designs.

**Figure 2.**
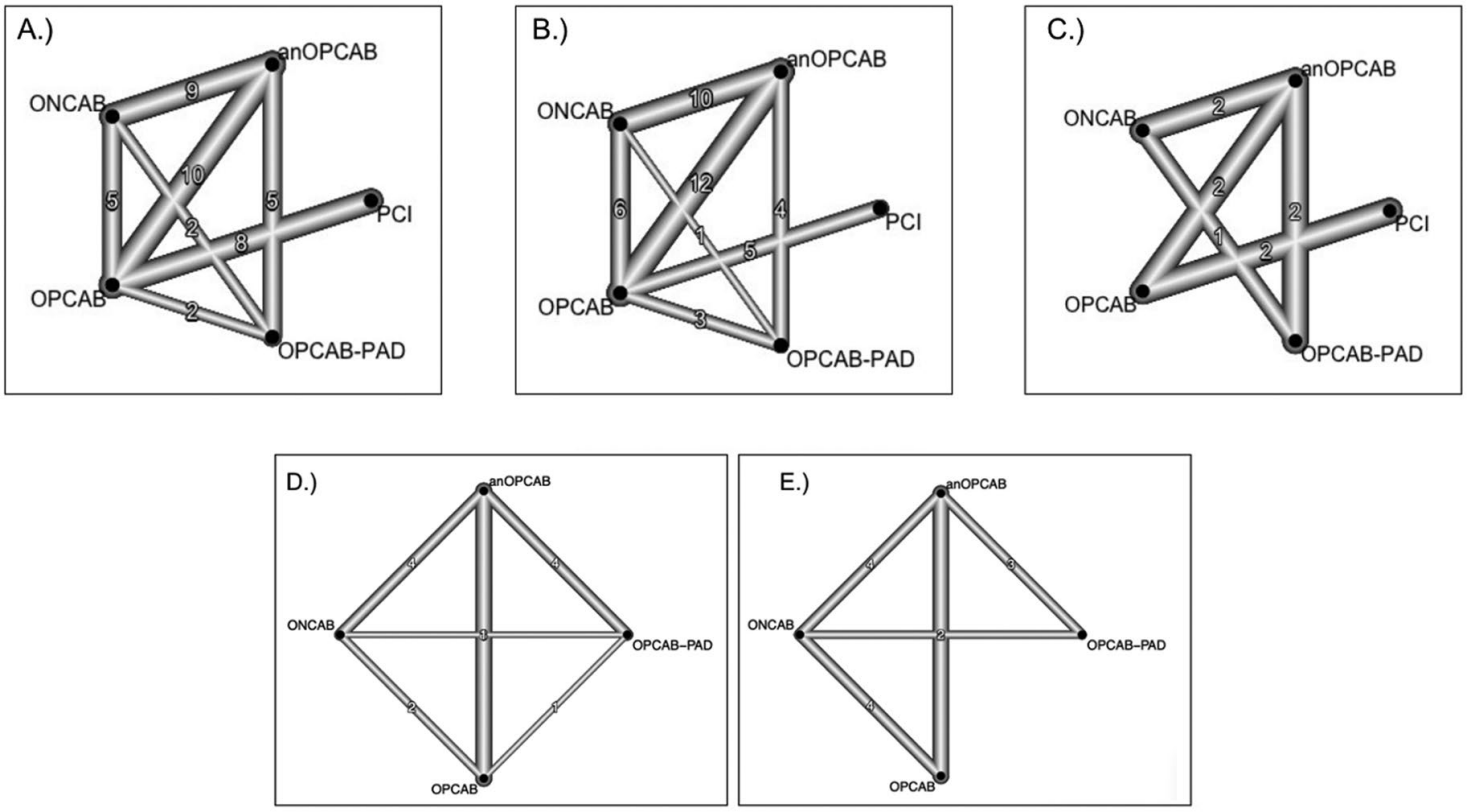
Network diagrams illustrating the connections among the five revascularization techniques: ONCAB, OPCAB, OPCAB-PAD, anOPCAB, and PCI. The thickness of the edges represents the relative number of studies comparing each pair of interventions. (A) Network structure for early mortality outcomes. (B) Network structure for early stroke outcomes. (C) Network structure for complete revascularization outcomes. (D) Network structure for POAF outcomes. (E) Network structure for renal failure outcomes.

## Outcomes

### Early mortality

**Figure 3.**
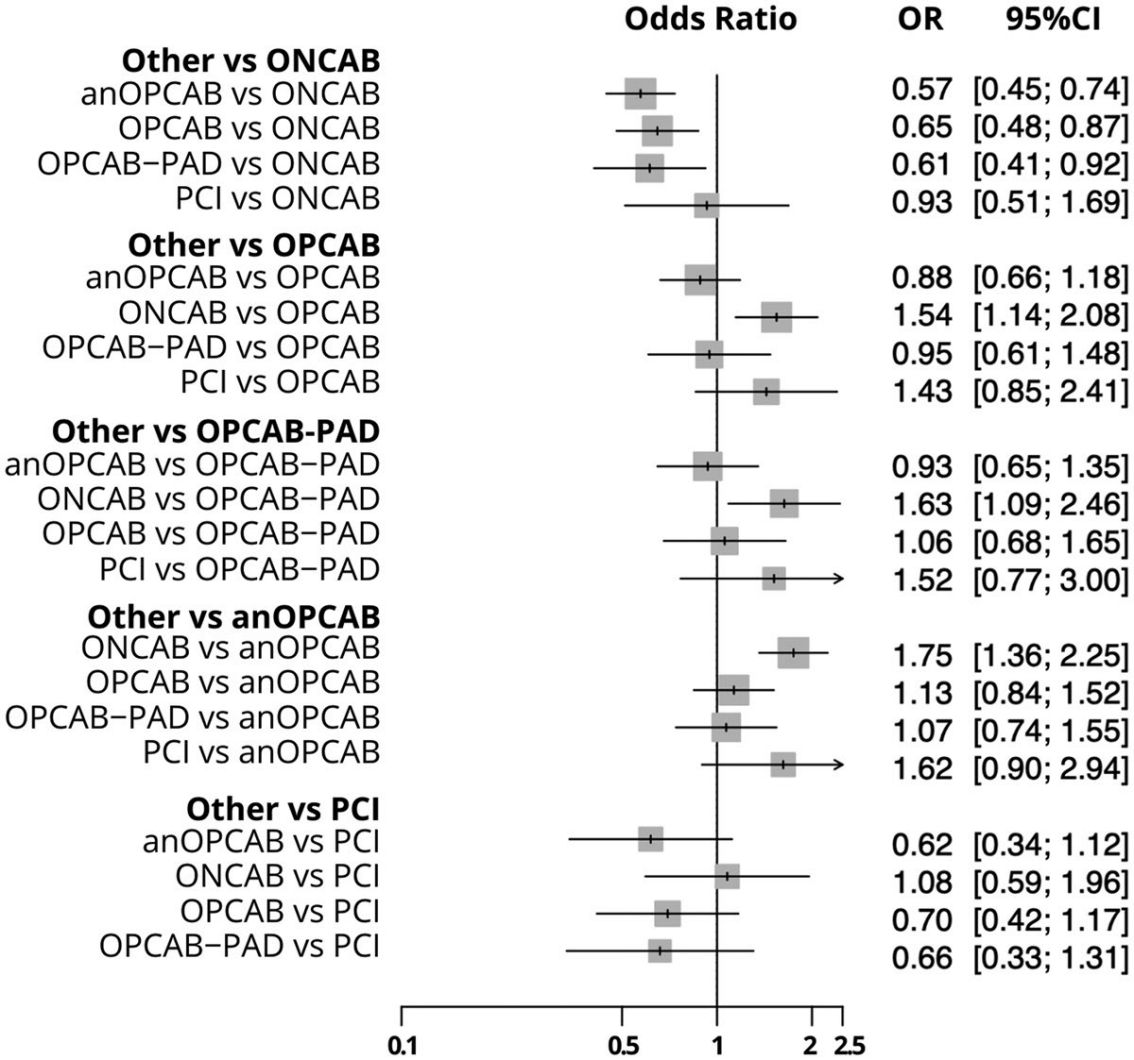
Combined Forest plot for 30-Day mortality comparing different coronary revascularization techniques. This forest plot displays the odds ratios (or) with 95% confidence intervals (CI) for 30-day mortality across various coronary revascularization strategies. ONCAB: on-pump coronary artery bypass; OPCAB: off-pump coronary artery bypass; OPCAB-PAD: OPCAB with proximal anastomotic device; anOPCAB: anaortic OPCAB; PCI = percutaneous coronary intervention.

A total of 27 studies [12–38] with 41 pairwaise comparisons (Figure 2A) were included in the analysis of early mortality, comparing five interventions: anOPCAB, OPCAB-PAD, OPCAB, ONCAB, and PCI. Compared to ONCAB, anOPCAB (OR: 0.57, 95% CI: 0.44–0.73, *p* < 0.001), OPCAB (OR: 0.64, 95% CI: 0.47–0.87, *p* = 0.004), and OPCAB-PAD (OR: 0.61, 95% CI: 0.40–0.92, *p* = 0.01) were associated with significantly lower early mortality. PCI, however, showed no significant difference compared to ONCAB (OR: 0.92, 95% CI: 0.51–1.68, *p* = 0.80). When PCI was used as reference, none of the comparisons, including anOPCAB, ONCAB, OPCAB, or OPCAB-PAD, were statistically significant. Heterogeneity for early mortality was low (*I*^2^ = 14.7%), with no significant inconsistency between designs (*p* = 0.95). The detailed comparisons are presented in the combined forest plot for early mortality (Figure 3).

### Early stroke

A total of 26 studies [13–33,38–43] with 41 comparisons (Figure 2B) were included in the analysis of early stroke. Using ONCAB as the reference, anOPCAB (OR: 0.29, 95% CI: 0.21–0.40, *p* < 0.001), OPCAB (OR: 0.61, 95% CI: 0.46–0.82, *p* = 0.001), and OPCAB-PAD (OR: 0.32, 95% CI: 0.21–0.49, *p* < 0.001) were associated with significantly lower rates of early stroke compared to ONCAB. PCI also showed a reduced stroke risk compared to ONCAB (OR: 0.14, 95% CI: 0.06–0.31, *p* < 0.001).

**Figure 4.**
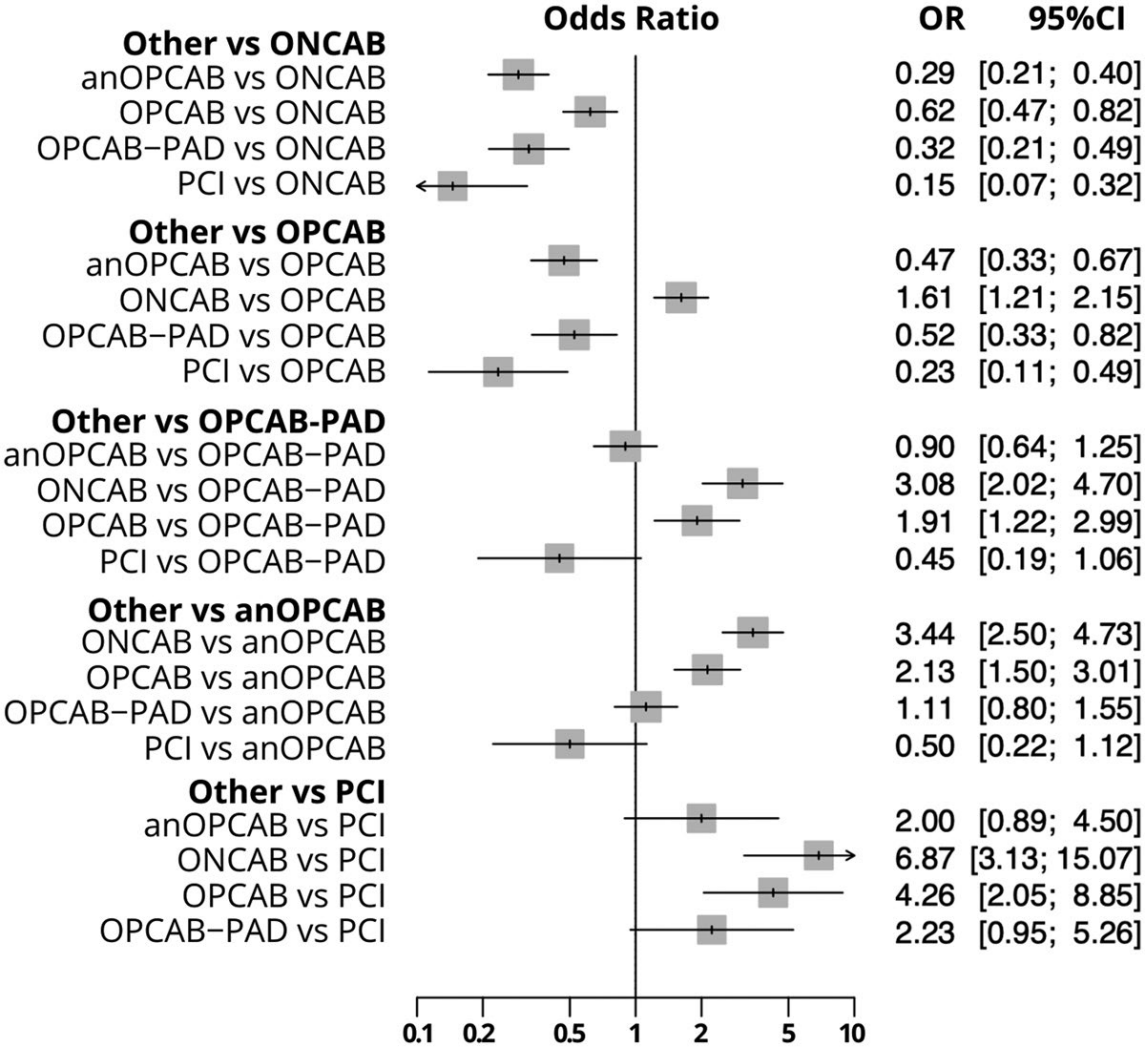
Combined Forest plot for early stroke comparing different coronary revascularization techniques. This forest plot displays the odds ratios (or) with 95% confidence intervals (CI) for early stroke across various coronary revascularization strategies. ONCAB: on-pump coronary artery bypass; OPCAB: off-pump coronary artery bypass; OPCAB-PAD: OPCAB with proximal anastomotic device; anOPCAB: anaortic OPCAB; PCI: percutaneous coronary intervention.

Compared to OPCAB, anOPCAB (OR: 0.47, 95% CI: 0.33–0.66, *p* < 0.001), OPCAB-PAD (OR: 0.52, 95% CI: 0.33–0.82, *p* = 0.004) and PCI (OR: 0.23, 95% CI: 0.11–0.48, *p* < 0.001) were significantly associated with lower stroke risks. When using anOPCAB as reference, both of ONCAB (OR: 3.4, 95% CI: 2.49–4.72, *p* < 0.001) and OPCAB (OR: 2.12, 95% CI: 1.50–3.01, *p* < 0.001) were associated with a higher stroke risk. Heterogeneity for early stroke was minimal (I^2^ = 0.7%), and tests for heterogeneity and inconsistency showed no significant differences between designs (*p* = 0.4578). The detailed comparisons are presented in the combined forest plot for early stroke (Figure 4).

### Complete revascularization

**Figure 5.**
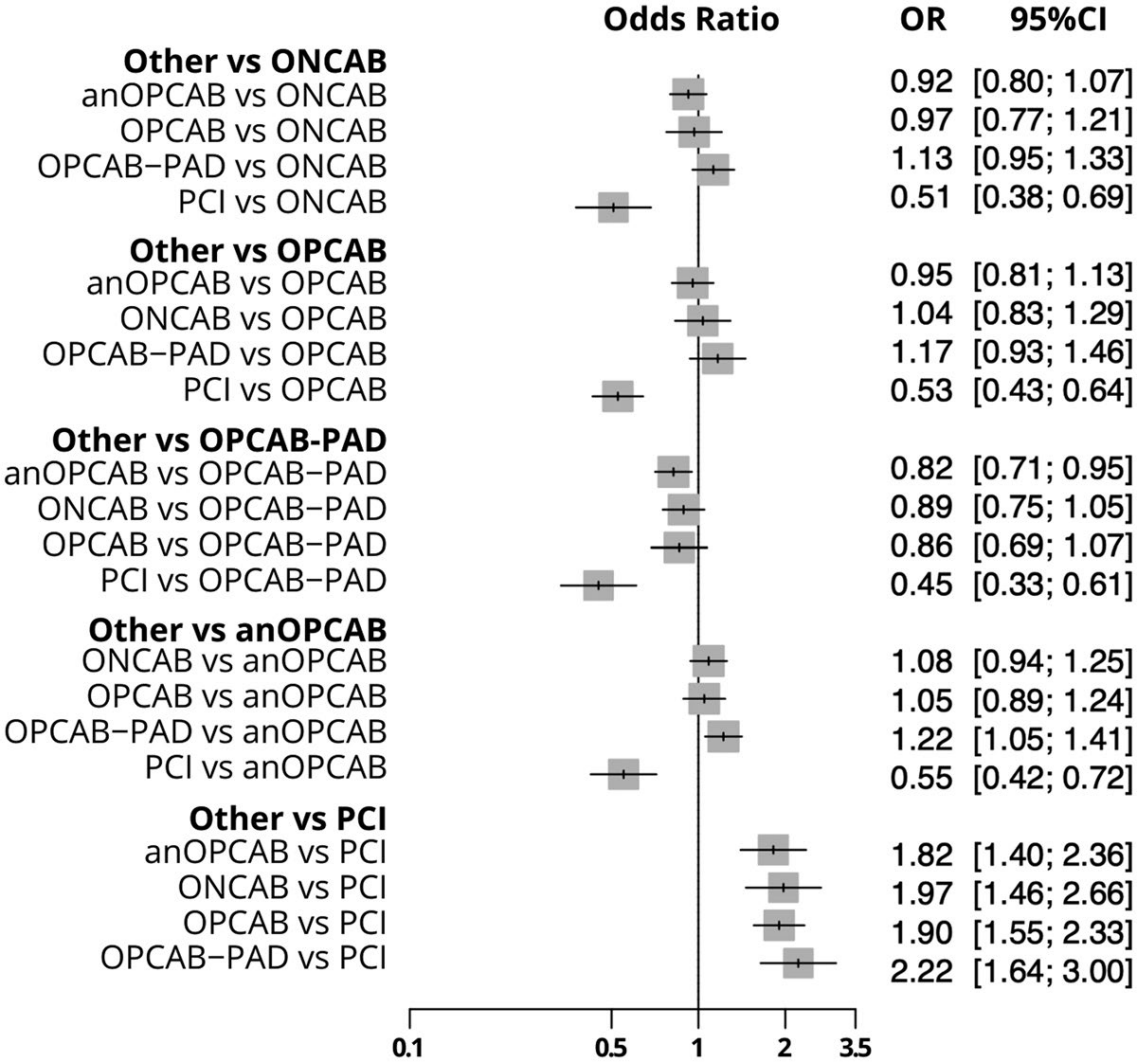
Combined Forest plot for complete revascularization across coronary revascularization techniques. This plot displays the odds ratios (or) with 95% confidence intervals (CI) for achieving complete revascularization among different coronary revascularization methods. ONCAB: on-pump coronary artery bypass; OPCAB: off-pump coronary artery bypass; OPCAB-PAD: OPCAB with proximal anastomotic device; anOPCAB: anaortic OPCAB; PCI: percutaneous coronary intervention.

In the analysis of seven studies [14,15,17,23,29,32,35] with nine pairwise comparisons (Figure 2C), PCI was consistently associated with a lower likelihood of achieving complete revascularization compared to ONCAB (OR: 0.50, 95% CI: 0.37–0.68, *p* < 0.001), OPCAB (OR: 0.52, 95% CI: 0.42–0.64, *p* < 0.001), OPCAB-PAD (OR: 0.45, 95% CI: 0.33–0.60, *p* < 0.001), and anOPCAB (OR: 0.55, 95% CI: 0.42–0.71, *p* < 0.001). Comparisons between anOPCAB, OPCAB, ONCAB, and OPCAB-PAD did not show significant differences, except for OPCAB-PAD versus anOPCAB, where OPCAB-PAD showed a higher likelihood of complete revascularization (OR: 1.22, 95% CI: 1.05–1.41, *p* = 0.007). High heterogeneity (*I*^2^ = 90.2%) was observed, with significant inconsistency between designs (*p* = 0.0141). The detailed comparisons are presented in the combined forest plot (Figure 5).

### Postoperative atrial fibrillation (POAF)

**Figure 6.**
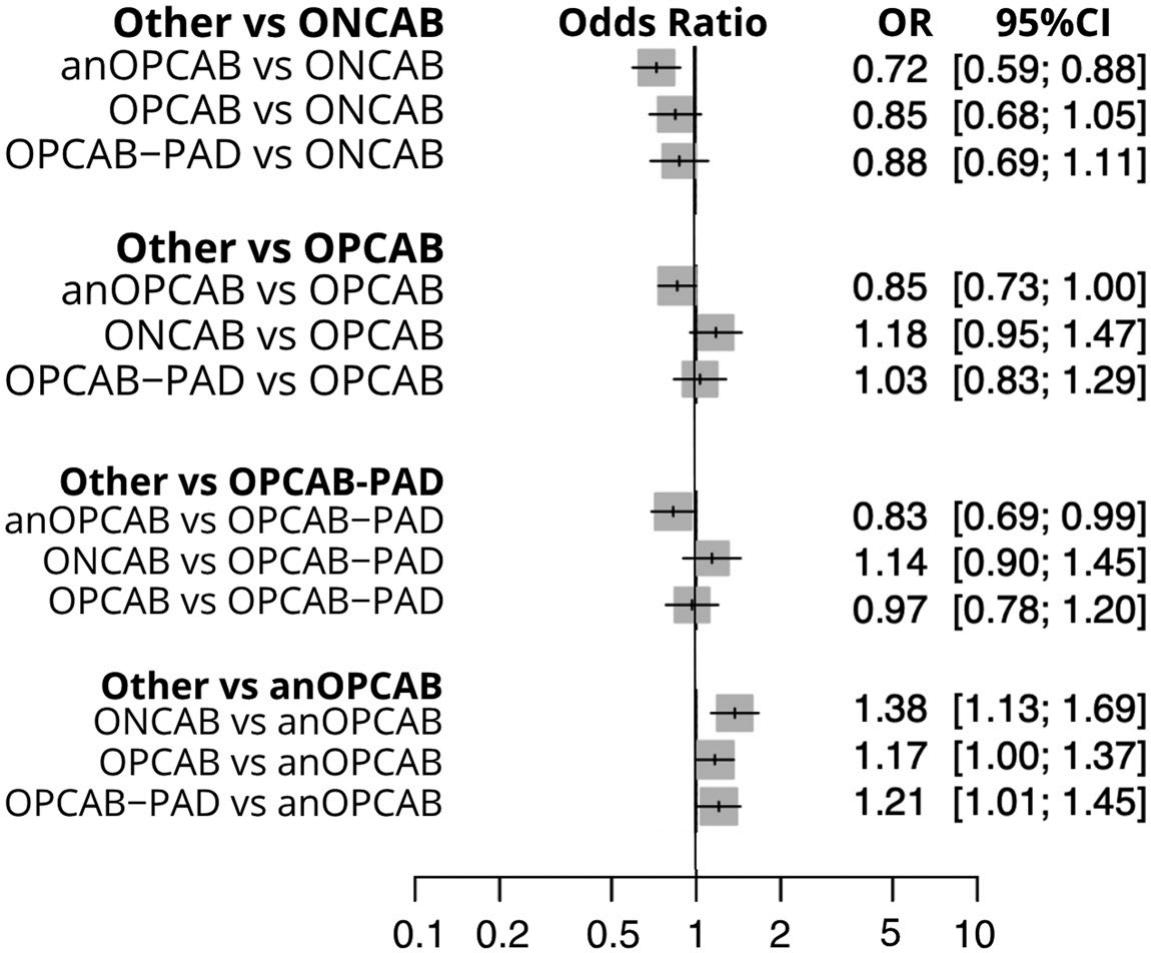
Combined Forest plot for postoperative atrial fibrillation (POAF) across coronary revascularization techniques. This plot displays the odds ratios (or) with 95% confidence intervals (CI) for POAF among different coronary revascularization methods. ONCAB: on-pump coronary artery bypass; OPCAB: off-pump coronary artery bypass; OPCAB-PAD: OPCAB with proximal anastomotic device; anOPCAB: anaortic OPCAB; PCI: percutaneous coronary intervention.

Twelve studies were included in the analysis of POAF outcomes [14,15,17,20,21,23–26,29,31,41]. When using anOPCAB as the reference, the risk of POAF was significantly lower compared to ONCAB (OR 0.72; 95% CI: 0.59–0.88; *p* = 0.0015) and OPCAB-PAD (OR 0.83; 95% CI: 0.69–0.99; *p* = 0.0398), and marginally lower than OPCAB (OR 0.85; 95% CI: 0.73–1.00; *p* = 0.05). In comparison, OPCAB-PAD did not differ significantly from ONCAB (OR 0.88; 95% CI: 0.69–1.11; *p* = 0.2804) or OPCAB (OR 1.03; 95% CI: 0.83–1.29; *p* = 0.7644). Similarly, POAF risk was comparable between OPCAB and ONCAB (OR 0.85; 95% CI: 0.68–1.05; *p* = 0.1303). Moderate heterogeneity was observed (I^2^ = 61.1%), accompanied by significant inconsistency between designs (*p* = 0.0002). The detailed comparisons are presented in the combined forest plot (Figure 6).

### Renal failure

**Figure 7.**
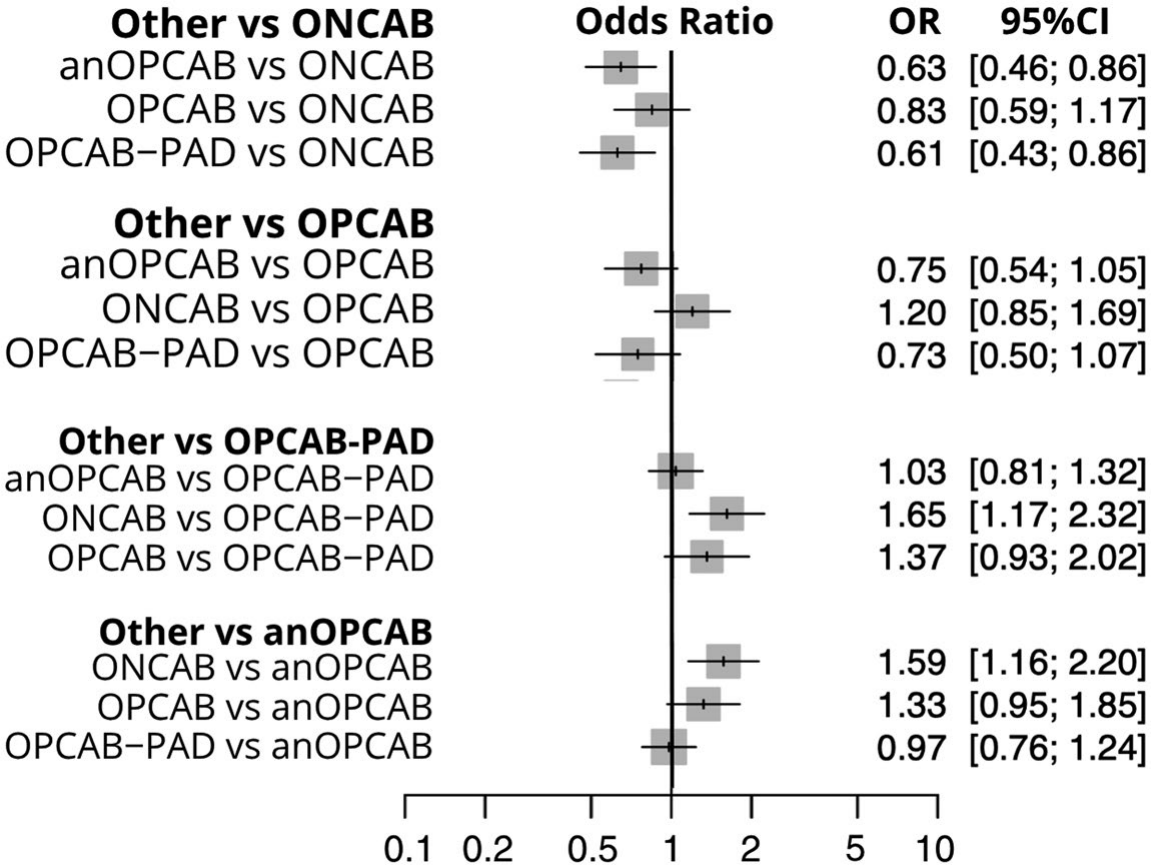
Combined Forest plot for postoperative renal failure across coronary revascularization techniques. This plot displays the odds ratios (or) with 95% confidence intervals (CI) for renal failure among different coronary revascularization methods. ONCAB: on-pump coronary artery bypass; OPCAB: off-pump coronary artery bypass; OPCAB-PAD: OPCAB with proximal anastomotic device; anOPCAB: anaortic OPCAB; PCI: percutaneous coronary intervention.

A total of 13 studies included in the analysis [15–21,23–25,29–31]. Renal failure outcomes favored anOPCAB and OPCAB-PAD over ONCAB. Compared to ONCAB, both anOPCAB (OR: 0.63, 95% CI: 0.46–0.86; *p* = 0.004) and OPCAB-PAD (OR: 0.61, 95% CI: 0.43–0.86; *p* = 0.004) were associated with significantly lower risk of postoperative renal failure. Other comparisons, including anOPCAB vs OPCAB (OR: 0.75, 95% CI: 0.54–1.05), did not reach statistical significance. The analysis revealed low heterogeneity (*I*^2^ = 0%) and no significant within-design heterogeneity, although a modest inconsistency was observed between designs (*Q* = 6.00, *p* = 0.0497). The detailed comparisons are presented in the combined forest plot (Figure 7).

### P-score rankings across outcomes

P-score analysis was conducted to rank the effectiveness of each coronary revascularization strategy across five outcomes: early mortality, early stroke, complete revascularization, postoperative atrial fibrillation (POAF), and renal failure. For early mortality, anOPCAB achieved the highest P-score (0.8449), followed by OPCAB-PAD (0.7079), OPCAB (0.6300), PCI (0.2135), and ONCAB (0.1037). These rankings support the mortality benefit of off-pump and anaortic approaches. In terms of early stroke, anOPCAB again ranked first (0.9915), followed by OPCAB-PAD (0.6619), OPCAB (0.4824), PCI (0.2778), and ONCAB (0.0863), emphasizing the stroke-sparing effect of avoiding or minimizing aortic manipulation. For complete revascularization, OPCAB-PAD led with a P-score of 0.9565, followed by OPCAB (0.8021), anOPCAB (0.7414), ONCAB (0.5000), and PCI (0.0000). These results highlight the superiority of surgical techniques in achieving full revascularization.

In POAF prevention, anOPCAB had the highest P-score (0.9847), substantially outperforming OPCAB-PAD (0.4206), OPCAB (0.5259), and ONCAB (0.0687). This reinforces the role of anaortic approaches in reducing atrial arrhythmias. For renal failure, OPCAB-PAD ranked highest (0.8471), followed closely by anOPCAB (0.7846), with OPCAB (0.3175) and ONCAB (0.0508). These rankings suggest that avoiding cardiopulmonary bypass and limiting aortic manipulation may contribute to better renal outcomes.

### Publication bias assessment

**Figure 8.**
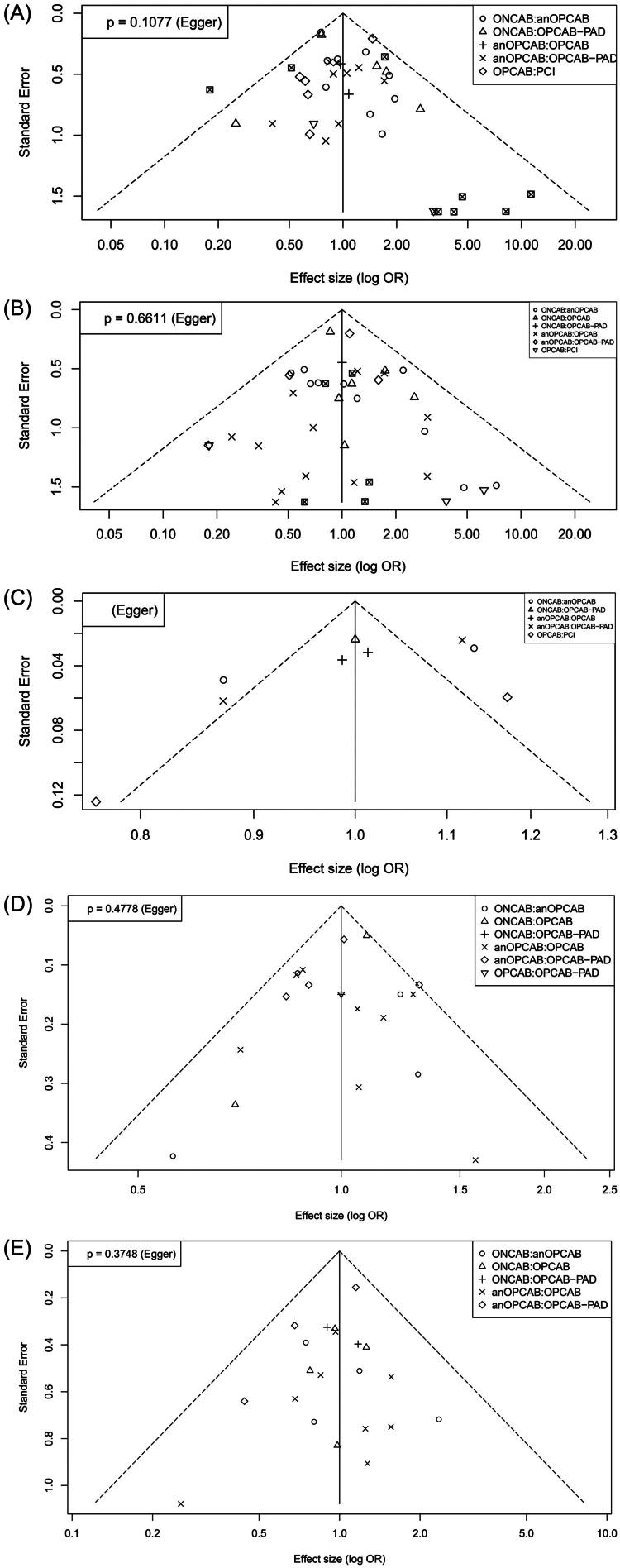
Funnel plots publication bias assessment for: (a) Early mortality; (B) Early stroke and (C) Complete revascularization; (D) Postoperative atrial fibrillation; (E) Renal failure.

Funnel plots and Egger’s regression test were used to assess potential publication bias for early mortality, early stroke, complete revascularization, postoperative atrial fibrillation (POAF), and renal failure outcomes (Figure 8).

For early mortality, the funnel plot appeared visually symmetrical. Egger’s test yielded a p-value of 0.1077, with a bias estimate of 0.4782 (SE: 0.2904), indicating no significant evidence of publication bias.

For early stroke, the funnel plot also appeared symmetrical, and Egger’s test showed a p-value of 0.6611 (bias estimate = 0.1053; SE = 0.2384), suggesting no evidence of publication bias.

For complete revascularization, Egger’s test returned a *p*-value of 0.1606, with a bias estimate of 0.4197 (SE = 0.2878). While statistically non-significant, the funnel plot suggested some asymmetry. Given the high heterogeneity (*I*^2^ = 90.2%), caution is warranted when interpreting this result.

For POAF, Egger’s test yielded a p-value of 0.4778, with a bias estimate of −0.3629 (SE = 0.5005), indicating no significant publication bias. Despite moderate heterogeneity (*I*^2^ = 61.1%), the funnel plot appeared reasonably symmetrical.

For renal failure, Egger’s test showed a *p*-value of 0.3748, with a bias estimate of −0.2996 (SE = 0.3296), suggesting no evidence of publication bias. Heterogeneity was low (*I*^2^ = 0%), and the funnel plot demonstrated visual symmetry.

In summary, no significant publication bias was detected across all outcomes, although the combination of high heterogeneity and visual funnel plot asymmetry for complete revascularization suggests the need for cautious interpretation of that specific outcome.

## Discussion

This network meta-analysis compared five revascularization techniques: ONCAB, OPCAB, OPCAB-PAD, anOPCAB, and PCI. A key strength of this approach lies in its ability to generate indirect comparisons between all five procedures, providing comprehensive insights that are not feasible in pairwise meta-analyses.

Surgical techniques consistently outperformed PCI in achieving complete revascularization, a finding aligned with established trials such as SYNTAX, which emphasized the importance of full anatomical revascularization in improving prognosis among patients with complex coronary anatomy [44]. While PCI remains a less invasive option with faster recovery, its limitations in achieving durable and complete revascularization, especially in diffuse or multi-vessel lesions, underscore the continued role of surgery in suitable candidates.

Among surgical options, anOPCAB consistently demonstrated favorable outcomes, ranking highest in stroke prevention, survival, and POAF reduction. The elevated P-scores across these outcomes underscore the benefit of avoiding both cardiopulmonary bypass and aortic manipulation, especially in patients at high embolic or neurologic risk. Similarly, OPCAB-PAD emerged as a strong performer, particularly in complete revascularization, supported by the highest P-score in that domain. This likely reflects the technical advantages of proximal anastomotic devices, which facilitate full revascularization while minimizing procedural risks. The favorable outcomes observed with OPCAB-PAD may be attributed to both the minimization of aortic manipulation and the consistent performance of proximal anastomotic devices (PAD), such as the Heartstring and Enclose II systems. These devices enable bloodless proximal anastomoses without the need for aortic clamping, an established risk factor for perioperative stroke. Their design also allows multiple proximal grafts to be constructed through simple repositioning, improving surgical efficiency in complex multivessel procedures [29]. The EPIC trial demonstrated that the PAS-Port proximal anastomotic device enables the construction of effective proximal anastomoses, offering a viable alternative that may enhance procedural efficiency by reducing the time required for anastomosis construction [45]. This combination of cerebral protection and technical practicality supports the growing role of OPCAB-PAD in modern coronary revascularization strategies.

However, the superior revascularization outcomes associated with surgical techniques must be weighed against their potential procedural risks. While our pooled data indicate lower rates of postoperative atrial fibrillation (POAF) and renal failure with anOPCAB compared to ONCAB, other important complications such as bleeding, infection, and broader perioperative risks were not assessed in this analysis. These safety considerations remain crucial in clinical decision-making. Therefore, the choice of revascularization strategy should be individualized, balancing anatomical complexity with each patient’s comorbidities and risk tolerance.

The comparable performance of OPCAB and ONCAB in terms of early mortality and complete revascularization underscores the effectiveness of off-pump techniques as a viable alternative to traditional on-pump methods. This finding is consistent with results from the CORONARY trial, which reported similar long-term outcomes between OPCAB and ONCAB, despite the reduced use of cardiopulmonary bypass in the former [46,47].

While our study shows that PCI is limited in achieving complete revascularization, we acknowledge that we did not assess long-term outcomes such as target lesion failure or mortality beyond 30 days. Therefore, any conclusions regarding PCI’s long-term efficacy should be interpreted cautiously. Nonetheless, our findings reinforce that in patients for whom long-term vessel patency and full anatomical correction are priorities, surgical options particularly those minimizing aortic manipulation remain central.

Notably, the inclusion of POAF and renal failure outcomes provides a broader view of procedural safety. The consistency in findings across studies for early mortality, stroke, and renal outcomes strengthens the reliability of our conclusions. For complete revascularization, we observed both high heterogeneity and funnel plot asymmetry, although Egger’s test did not reach statistical significance. This combination raises concern for potential small-study effects or selective publication, which may bias the pooled effect estimate. Furthermore, the lack of standardized definitions across studies and possible variations in angiographic assessment or surgical reporting could further contribute to this inconsistency. These findings highlight the importance of interpreting the complete revascularization outcome with caution, and future studies should aim for more consistent definitions and reporting standards to improve comparability. For POAF, moderate inconsistency was noted. This likely reflects differences in perioperative management strategies and baseline patient characteristics, such as age or left atrial size, which were not uniformly reported across studies. We acknowledge that subgroup analyses (e.g. based on study design, geographical setting, or publication year) could help identify the source of heterogeneity. However, the number of studies per subgroup was insufficient to perform robust subgroup meta-analysis or meta-regression without risking statistical underpowering and false inferences. Despite this limitation, we took steps to ensure transparency and robustness in the analysis. We evaluated heterogeneity using the *I*^2^ statistic and provided detailed forest plots for each outcome to allow visual inspection. We have also expanded the explanation of this issue in the Limitations section, recognizing the importance of standardized definitions and methodological consistency in future research. Additionally, we acknowledge that potential unmeasured confounders, including comorbidities, surgical expertise, or hospital volume, may also contribute to variability. These were often not consistently reported and thus could not be adjusted for in our analysis.

Overall this analysis supports the growing role of anOPCAB and OPCAB-PAD as refined surgical strategies that may offer neuroprotection and reduce perioperative morbidity, particularly in high-risk patients. These approaches offer compelling alternatives to traditional on-pump surgery, with evolving technologies continuing to enhance their safety profile.

### Limitation

This network meta-analysis provides a comprehensive comparison of established coronary revascularization strategies using both direct and indirect evidence. However, several limitations must be acknowledged. First, although we included only studies involving multivessel disease, heterogeneity in study designs, surgical expertise, and perioperative care protocols may have influenced the outcomes particularly for complete revascularization, which showed high heterogeneity (*I*^2^ = 90.2%). Second, our analysis focused primarily on short-term endpoints (e.g. early mortality, stroke, POAF, and renal failure), and did not evaluate long-term outcomes such as repeat revascularization, or survival beyond 30 days. Third, other clinically important endpoints including bleeding, infection, and duration of hospital stay were not consistently reported across studies and therefore could not be assessed.

Additionally, the analysis relied on study-level data rather than individual patient data, limiting our ability to perform subgroup analyses based on age, comorbidities, or anatomical complexity. The lack of standardized definitions across studies, particularly for complete revascularization and postoperative complications, may also have introduced variability. While publication bias was not detected for most outcomes, some asymmetry observed in the funnel plot for complete revascularization warrants cautious interpretation. Future research should aim for standardized reporting, inclusion of long-term endpoints, and individual patient-level analyses to provide more nuanced, personalized guidance for clinical decision-making.

## Conclusion

This network meta-analysis highlights that off-pump surgical techniques particularly anaortic OPCAB (anOPCAB) are associated with the lowest risk of postoperative stroke, while OPCAB and OPCAB-PAD demonstrate comparable early mortality and revascularization success to conventional ONCAB. Although PCI offers a less invasive alternative with favorable neurological outcomes, its limited capacity for achieving complete revascularization restricts its role in the management of complex multivessel disease. These findings support the use of tailored surgical approaches especially anOPCAB and OPCAB-PAD for patients with MVD who are suitable surgical candidates, balancing procedural risk with the need for complete and durable revascularization.

## Supplementary Material

Supplemental Material.docx

## Data Availability

The datasets generated and/or analyzed during the current study are available from the corresponding author upon reasonable request. The original contributions presented in this study are included in the article.

## Appendix 1. Search strategy per database

Pubmed Search Strategy:

(‘Coronary Artery Bypass’[Mesh] OR ‘Coronary Artery Bypass, Off-Pump’[Mesh] OR ‘Coronary Artery Bypass Surgery’ OR ‘OPCAB’ OR ‘anaortic OPCAB’ OR ‘OPCAB-PAD’ OR ‘proximal anastomotic device’)

AND

(‘Percutaneous Coronary Intervention’[Mesh] OR ‘PCI’)

AND

(‘early mortality’ OR ‘stroke’ OR ‘complete revascularization’)

ScienceDirect Search Strategy

(‘coronary artery bypass’ OR ‘off-pump coronary artery bypass’ OR ‘OPCAB’ OR ‘anaortic OPCAB’ OR ‘OPCAB-PAD’ OR ‘proximal anastomotic device’)

AND

(‘PCI’ OR ‘percutaneous coronary intervention’)

AND

(‘early mortality’ OR ‘stroke’ OR ‘complete revascularization’)

Embase (*via* Elsevier) Search Strategy

(‘coronary artery bypass’/exp OR ‘off pump coronary artery bypass’ OR OPCAB OR ‘anaortic OPCAB’ OR ‘OPCAB-PAD’ OR ‘proximal anastomotic device’)

AND

(‘percutaneous coronary intervention’/exp OR PCI)

AND

(‘early mortality’/exp OR ‘stroke’/exp OR ‘complete revascularization’/exp)

## Appendix 2

Quality assessment using the: A. ROBINS-I tool for observational studies. B. RoB2 tool for randomized studies.
